# A novel probiotics therapy for aging-related leaky gut and inflammation

**DOI:** 10.1093/geroni/igab046.2521

**Published:** 2021-12-17

**Authors:** Shaohua Wang, Bo Wang, Sidharth Mishra, Shalini Jain, Jingzhong Ding, Stephen Krtichevsky, Dalane Kitzman, Hariom Yadav

**Affiliations:** 1 Wake Forest School of Medicine, Winston-Salem, North Carolina, United States; 2 NC A&T State University, Greensboro, North Carolina, United States; 3 Wake Forest School of Medicine, Wake Forest School of Medicine, North Carolina, United States; 4 Wake Forest Medical Center, Winston-Salem, North Carolina, United States

## Abstract

Inflammaging characterized with increased low grade inflammation in older adults is common determinant of unhealthy aging; and is a major risk factor of morbidity and mortality in older adults. The precise origin of inflammation in older adults is not known, however, emerging evidence indicate that increased intestinal epithelial permeability (leaky gut) and abnormal (dysbiotic) gut microbiota could be one of the key source. However, no preventive and treatment therapies are available to reverse the leaky gut and microbiome dysbiosis in older adults. Here, we presented the evidence that a human-origin probiotics cocktail containing 5 Lactobacillus and 5 Enterococcus strains isolated from healthy human infant gut can ameliorate aging-related metabolic, physical and cognitive dysfunctions in older mice. We show that the Feeding this probiotic cocktail prevented high-fat diet–induced (HFD-induced) abnormalities in glycose metabolism and physical functions in older mice and reduced microbiota dysbiosis, leaky gut, inflammation. Probiotic-modulated gut microbiota reduced leaky gut by increasing tight junctions on intestinal epithelia, which in turn reduced inflammation. Mechanistically, probiotics increased bile salt hydrolase activity in older microbiota, which in turn increased taurine deconjugation from bile acids to increase free taurine abundance in the gut. We further show that taurine stimulated tight junctions and suppressed gut leakiness. Further, taurine increased life span, reduced adiposity and leaky gut, and enhanced physical function in Caenorhabditis elegans. Whether this novel human origin probiotic therapy could prevent or treat aging-related leaky gut and inflammation in the elderly by reversing microbiome dysbiosis requires evaluation.

